# MicroRNA-495 downregulates FOXC1 expression to suppress cell growth and migration in endometrial cancer

**DOI:** 10.1007/s13277-015-3686-6

**Published:** 2015-07-22

**Authors:** Yan-Ying Xu, Jing Tian, Quan Hao, Li-Rong Yin

**Affiliations:** 1 Department of Gynecology, The Second Hospital of Tianjin Medical University, Tianjin, China; 2 National Clinical Research Center of Cancer, Key Laboratory of Cancer Prevention and Therapy, Department of Gynecological Oncology, Tianjin Medical University Cancer Institute and Hospital, Tianjin, China

**Keywords:** Endometrial cancer, MicroRNA, FOXC1, Apoptosis, Oncogene

## Abstract

MicroRNAs (miRNAs) are a class of noncoding RNAs and function as key regulators of gene expression at the post-transcriptional level. In this study, we found that miR-495 reduces cell growth, induces apoptosis and suppresses the migration of endometrial cancer by directly inhibiting FOXC1 expression. Further analysis revealed that FOXC1 promotes growth and migration and functions as an oncogene in vitro. FOXC1 overexpression reversed the cellular responses mediated by miR-495 in endometrial cancer cells. We also found that miR-495 suppresses the growth of endometrial cancer in vivo. Altogether, these results indicate that miR-495 acts as a tumour suppressor gene by targeting FOXC1 at the post-transcriptional level in endometrial cancer.

## Introduction

MicroRNAs (miRNAs) are a group of highly conserved small noncoding RNAs and regulate target expression by complete or incomplete complementarity with messenger RNA of the target gene 3′UTR at the seed sequence to lead to either mRNA degradation or repression of translation [1]. Documented evidence has shown that miRNAs are involved in many cellular processes, including development, apoptosis, differentiation, metabolism and stress response, depending upon the regulation of specific target genes [1]. Meanwhile, miRNAs participate in the development of multiple diseases, including tumourigenesis [2]. The involvement of miRNAs in malignant tumours has been elucidated in multiple studies. Some miRNAs promote the process of tumourigenesis and function as oncogenes. For example, miR-106b is associated with a greater capacity for cell proliferation by the inhibition of G1-phase cell cycle arrest in melanoma cells [3]. In oesophageal squamous cell carcinoma, miR-9 promotes tumour metastasis by downregulating the expression of E-cadherin [4]. However, other miRNAs are expressed at a low level and act as tumour suppressors. MiR-27a can directly target KRAS to inhibit cell proliferation in oesophageal squamous cell carcinoma [5]. miR-99a suppresses the metastasis of human non-small cell lung cancer cells by targeting the AKT1 signalling pathway [6]. Recent studies have indicated that miRNAs are tissue- and disease-specific [7], which function and are expressed differently in many types of cancers. Thus, miRNAs can serve as powerful biomarkers, which are useful in the diagnosis and therapy of cancer [8].

In the tumour-associated miRNAs, the mechanism of miR-495 in cancer initiation and progression drew our attention, because miR-495 has been found to perform various biological functions in a variety of types of cancer. For instance, in breast cancer stem cells, miR-495, upregulated by E12/E47, promotes oncogenesis and hypoxia resistance via the downregulation of E-cadherin and REDD1 [9]. Li et al. found that miR-495 acts as a tumour suppressor and inhibits gastric cancer cell migration and invasion by regulating a novel member of the PTP family, PRL-3 [10, 11]. However, until now, the function and molecular mechanism of miR-495 in endometrial cancer remain largely unknown.

Endometrial cancer, the most prevalent cancer of the female genital tract, is the third most frequent gynaecologic malignancy worldwide. Approximately 42,160 cases were diagnosed with endometrial cancer, and 7780 women died of it in the USA in 2009 [12]. Worldwide, 226,000 women were diagnosed with endometrial cancer [13]. Previous studies have reported that endometrial cancer is a multifactorial disease with several aberrant steps in gene expression [14]. Several studies have indicated the alteration of miRNA expression in endometrial cancer, such as miR-101 [15], miR-205 [16], miR-204 [17] and miR-34a [18], miR-194 [19] and miR-130b [20], which are confirmed to be associated with the malignant phenotypes of endometrial cancer. In this study, we aimed to identify the novel biological function of miR-495 suggested by miRNA target prediction databases in endometrial cancer.

FOXC1 is a member of the forkhead box (FOX) transcription factor family and participates in a multitude of biological processes. Dysregulation of the FOXC1 protein contributes to carcinogenesis, including proliferation, apoptosis, differentiation, invasion and metastasis of several human cancers [21]. Muggerud et al. reported that FOXC1 knockdown mediates cell proliferation, migration and invasion inhibition in breast cancer cells [22]. In response to TGF-β, FOXC1 is upregulated transcriptionally and suppresses cell growth [23]. FOXC1 knockdown by siFOXC1 reduces the migration and invasion of HEC1A cells in endometrial cancer cells [17]. Here, we found that miR-495 was frequently downregulated, and FOXC1 was overexpressed in endometrial cancer tissues relative to normal tissues. miR-495 suppresses the growth of endometrial cancer cells by inducing cell apoptosis. miR-495 can also suppress the migration of endometrial cancer cells. Furthermore, FOXC1 was identified as a direct target of miR-495. Knockdown of FOXC1 suppressed, and overexpression of FOXC1 promoted the cell growth and migration of endometrial cancer in vitro. In vivo, we also examined the negative relationship between miR-495 and FOXC1 and the opposite effects of tumour growth. Taken together, our study is the first to document that miR-495 acts as a tumour suppressor gene by negatively regulating FOXC1 in endometrial cancer.

## Materials and methods

### Cell culture

The endometrial carcinoma cell lines AN3CA and KLE were obtained from ATCC and maintained in DMEM/F12 supplemented with 10 % FBS, 100 units/ml penicillin and 100 μg/ml streptomycin. The cells were incubated at 37 °C in a humidified chamber supplemented with 5 % CO2.

### Human tissue samples

Ten human endometrial cancer tissues and five normal tissues were obtained from the Tumor Bank Facility of Tianjin Medical University Cancer Institute and the National Foundation of Cancer Research (TBF of TMUCIH and NFCR) with the patients’ informed consent. All tissue stages of cancer were confirmed by pathology and immunohistochemistry, and samples were collected and frozen in liquid nitrogen and stored at 80 °C. The diagnoses of these samples were verified by pathologists. Approval for this work was granted by the Ethics Committee of Tianjin Medical University.

### RNA isolation and quantitative real-time (qRT)-PCR assay

Large and small RNAs from the tissue were isolated with mirVana miRNA Isolation Kit (Ambion, Austin, TX, USA) according to the manufacturer’s protocol. For RNA integrity assessment, part of an RNA sample was used for concentration and purity measurement (by A260 and A280 spectrophotometry), and another part of the sample was run on a 1.5 % denaturing agarose gel stained with ethidium bromide. A ratio of the absorbance at 260 and 280 nm (A260/280) of 1.8–2 was accepted. The sharp, clear 28S and 18S rRNA bands and the 2:1 ratio (28S:18S) were good indicators that the RNA was completely intact.

Five micrograms of an RNA sample was reverse-transcribed to cDNA using oligo (dT) primers and M-MLV reverse transcriptase (Promega, Madison WI); the cDNA was used for the amplification of FOXC1 and β-actin. Quantitative real-time PCR (qRT-PCR) was performed to detect the relative transcript levels of miR-495 and FOXC1. PCR was performed under the following conditions: 94 °C for 4 min was followed by 40 cycles of 94 °C for 1 min, 56 °C for 1 min and 72 °C for 1 min.

To detect the mature miR-495 levels, a stem-loop RT-PCR assay was performed using specific RT and PCR primers. U6 snRNA was used as an endogenous control. Five micrograms of total RNA was reverse-transcribed to cDNA with a specific RT primer that could fold into a stem-loop structure. The PCR cycles were as follows: 94 °C for 4 min was followed by 40 cycles of 94 °C for 30 s, 50 °C for 30 s and 72 °C for 30 s. PCR was performed using SYBR Premix Ex Taq Kit (TaKaRa, Madison, WI) according to the manufacturer’s instructions and analysed using 7300 RT-PCR system (ABI). The relative expression levels of the gene of interest were calculated by the 2^−ΔΔCt^ method. All primers were synthesised by AuGCT Inc. (Beijing, China).

### Cell transfection

One day before transfection, the cells were plated in a 48-well or 24-well plate. The transfections were performed using Lipofectamine 2000 Reagent according to the manufacturer’s protocol (Invitrogen, Carlsbad, CA, USA). The plasmids were used at a final concentration of 5 ng/l. To determine the transfection efficiency, we transfected either pcDNA3.1-EGFP or the negative control, pcDNA3.1, into the two cell lines and assessed the level of enhanced green fluorescent protein (EGFP) expression 48 h after transfection using fluorescence microscopy.

### Plasmid construction

The pSilencer/shRNA- FOCX1 (siFOCX1) vector was obtained by annealing two single-stranded complementary sequences that contained *Bam*HI and *Hin*dIII restriction sites at the ends. The fragment was then cloned into the pSilencer2.1/neo vector (Ambion) between the *Bam*HI and *Hin*dIII sites.

The pcDNA3.1 vector was used to generate a FOCX1-overexpression plasmid. The full-length human FOCX1 cDNA sequence (GenBank TM, NM_001453.2) was amplified by PCR using cDNA isolated from foetal brain tissue as the template. The FOCX1 gene was inserted into the *Eco*RI and *Xba*I restriction sites.

The EGFP expression vector (pcDNA3.1/EGFP) was constructed as previously described. The 3′UTR fragment of the FOCX1 gene containing the predicted miR-495 binding site was amplified by PCR using the primers. The PCR products were cloned into the pcDNA3.1/EGFP plasmid between the *Bam*HI and *Eco*RI restriction sites, and the resulting vector was named pcDNA3.1/EGFP- FOCX1 3′UTR. All of the inserted DNAs mentioned above were verified by DNA sequencing.

### Target prediction

TargetScan, PicTar and miRBase were used to predict the putative targets of miR-495.

### EGFP reporter assay

The 3′UTR segments of FOCX1 containing putative binding sites for miR-495 were inserted into pcDNA3.1/EGFP (pcDNA3.1/EGFP- FOCX1-UTR 648, 607 and 1629). The endometrial cancer cells AN3CA and KLE were cotransfected with pcDNA3.1/pri-miR-495 and pcDNA3.1/EGFP- FOCX1-UTR or with pcDNA3.1 negative control vector in 48-well plates. The vector pDsRed2-N1 (Clontech, USA) expressing red fluorescent protein (RFP) was used for normalisation. Approximately 48 h after transfection, the fluorescence intensity was measured with an F-4500 fluorescence spectrophotometer (Hitachi, Tokyo, Japan). The EGFP expression was normalised to RFP expression for each sample. Each experiment was repeated at least three times.

### Western blot

The transfected cells were washed with phosphate buffered saline (PBS) and lysed with radioimmunoprecipitation assay buffer (150 mM NaCl, 1 % Nonidet P-40, 1 % Triton X-100, 1 mM MgCl2, 0.1 % SDS, 10 Mm Tris-HCl, pH 7.4) for 30 min at 48 h after transfection. Cell extracts were cleared by centrifugation at 12,000×*g* for 10 min at 4 °C, and the supernatant was used for western blot analyses. All proteins were resolved on a 10 % SDS denaturing polyacrylamide gel and then transferred onto a nitrocellulose membrane. Membranes were incubated with blocking buffer for 90 min at room temperature and were then incubated overnight at 4 °C with a rabbit polyclonal anti-FOCX1 antibody (1:200; Tianjin Saier Biotech, Tianjin, China) or anti-glyceraldehyde phosphate dehydrogenase (GAPDH) antibody (1:200; Tianjin Saier Biotech, Tianjin, China) prepared in blocking buffer. The membranes were washed and incubated with a horseradish peroxidase (HRP)-conjugated secondary antibody (1:1000; Tianjin Saier Biotech, Tianjin, China). Protein expression was assessed by enhanced chemiluminescence and exposure to chemiluminescent film. LabWorks Image Acquisition and Analysis Software (UVP) was used to quantitate band intensities.

### Cell viability assay

Twenty-four hours after transfection, cells were seeded in 96-well plates at either 6*103 cells/well (AN3CA cells) or 15*103 cells/well (KLE cells). The MTT assay was used to measure cell viability at 24, 48 and 72 h after being seeded. The cells were incubated with MTT (at a final concentration of 0.5 mg/ml) at 37 °C for another 4 h. Then, the medium was removed, and the precipitated formazan was dissolved in 100 μl of dimethyl sulfoxide (DMSO). After shaking for 15 min, the absorbance at 570 nm (A570) was detected using a μQuant universal microplate spectrophotometer (BioTek Instruments, Winooski, VT).

### Colony formation assay

Cells were counted and seeded (300 cells/well) in 12-well plates (in triplicate). Fresh culture medium was replaced every three days. Colonies were counted only if they contained more than 50 cells, and the number of colonies was counted either 12 days (AN3CA cells) or 15 days (KLE cells) after seeding. The cells were stained using crystal violet. Colony formation was calculated by the colony formation number.

### Flow cytometry analysis of cell apoptosis

To measure apoptosis, cells were collected, washed with PBS and stained with fluorescein isothiocyanate-labelled annexin V (Invitrogen) and propidium iodide, and this was followed by flow cytometry analysis [24].

### In vitro migration assays

In vitro cell migration assays were performed using transwell chambers (pore size of 8 uM; Costar, Corning, NY). Transfected cells were resuspended in serum-free medium, and 200 μl of the cell suspension (4*10^4^ cells) was added to the upper chamber. Complete medium was added to the bottom wells of the chambers. For the screen, the cells that had not migrated after either 7 h (AN3CA cells) or 18 h (KLE cells) were removed from the upper face of the filters using cotton swabs. After fixation and staining in a dye solution containing 0.1 % crystal violet and 20 % methanol, the cells that had adhered to the lower membrane of the inserts were counted. Images of three different fields (5*magnification) were taken for each membrane, and the number of migratory cells was counted. The mean of triplicate assays for each experimental condition was used.

### Murine xenograft model

Six-week-old female nude mice were purchased from the animal facilities of the Chinese Academy of Medical Sciences and were housed in the animal facilities of Tianjin Medical University as approved by the Institutional Animal Care and Use Committee. AN3CA cells and KLE cells were subcutaneously injected into the flanks of the nude mice. After 22 days, the mouse were sacrificed, and the tumours were harvested and then stored at −80 °C for subsequent analysis.

### Immunohistochemistry

Immunohistochemistry was performed according to previously described methods [25]. The sections were pre-treated using microwave irradiation and were then blocked and incubated with polyclonal rabbit anti-human FOXC1 antibodies (Saier Biotechnology). The staining intensity was then assessed.

### Statistical analysis

Data are expressed as the means ± SD. Statistical analyses were performed using a paired *t* test. A *p* value <0.05 was considered to be statistically significant. One representative experiment is shown in the duplicates or triplicates used for the statistical analysis.

## Results

### miR-495 suppresses cell growth and induces apoptosis in endometrial cancer

**Fig. 1:**
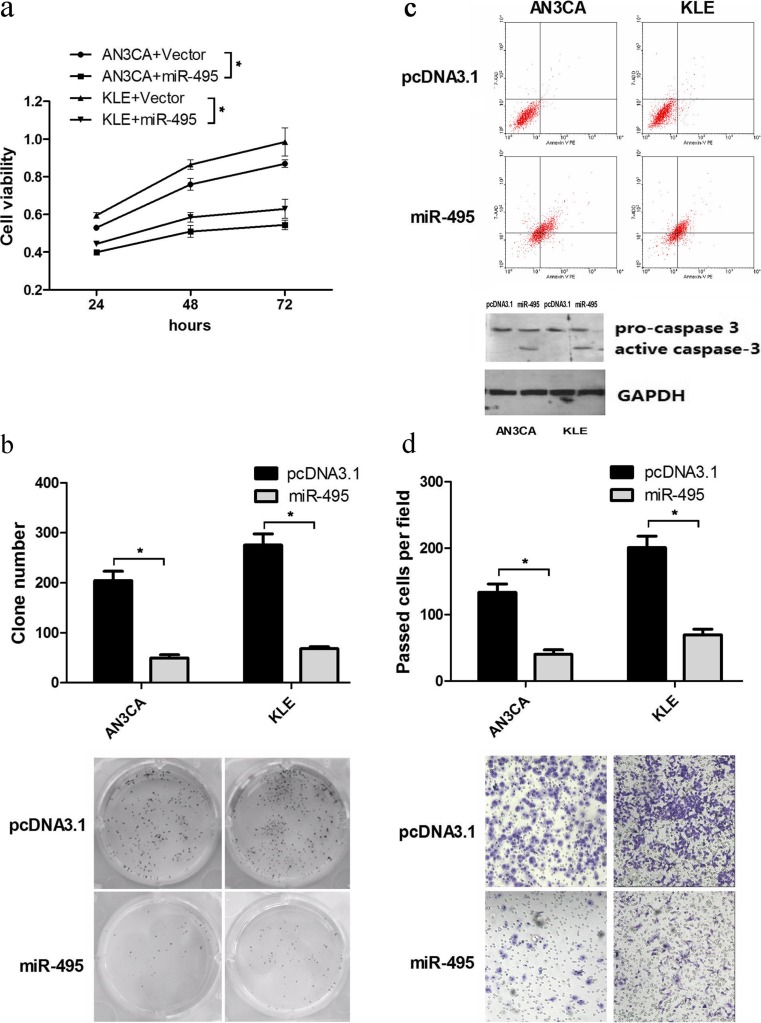
The functions of miR-495 in endometrial cancer cells, AN3CA and KLE cells. **a** The cell viability of AN3CA and KLE cells was determined by MTT assay 24, 48 and 72 h after transfection (*n* = 3,*p < 0.05). **b** Colony formation assay was employed to detect the effect of miR-495 on the cell growth activity of AN3CA and KLE cells (*n* = 3,*p < 0.05). **c** Flow cytometry analysis and western blot were used to detect cell apoptosis and caspase 3 in AN3CA and KLE cells

In order to investigate the effects of miR-495 in endometrial cancer, a miR-495 overexpression vector, pcDNA3.1/pri-miR-495 (miR-495), was constructed. Two endometrial cancer cell lines, AN3CA and KLE, were applied to examine the effects of miR-495 on cell viability, colony formation and apoptosis. The MTT assay was used to evaluate cell viability. As shown in Fig. 1a, the overexpression of miR-495 reduced the viability both in AN3CA and KLE cells at 24, 48 and 72 h post-transfection. To further determine the effect of miR-495 on long-term and independent cellular growth ability of AN3CA cells, we preformed the colony formation assay. The colony numbers in AN3CA cells transfected with miR-495 decreased by approximately 75.8 % (Fig. 1b). In KLE cells, the colony numbers were reduced by 75.3 % (Fig. 1b). To further explore the mechanism of miR-495 in reducing cell growth, we measured cell apoptosis of miR-495 in AN3CA and KLE cells. Flow cytometry (FCM) was used to examine the properties of cell apoptosis. As shown in Fig. 1c, the group of miR-495 overexpression had a higher property of apoptosis compared with the negative control in AN3CA and KLE cells. Meanwhile, we also detected the marker of cell apoptosis, active caspase 3 and pro-caspase 3. The results shown that both in AN3CA and KLE cells, caspase 3 activity was much higher in miR-495 group (Fig. 1c). These results indicated that cell growth inhibition by miR-495 is generated as a consequence of the promotion of cell apoptosis in vitro.

### miR-495 suppresses the properties of migration in vitro

Migration is one of the essential factors for metastasis, which is a dangerous property of cancer. To investigate the effect of miR-495 on the migration of endometrial cancer, a transwell assay without Matrigel was employed. In AN3CA cells, approximately 30 % of cells overexpressing miR-495 migrated to the basal side of the membrane compared to the negative control (Fig. 1d). Similarly, 34.6 % of KLE cells with miR-495 overexpression migrated to the basal side relative to the control cells (Fig. 1d). These data suggested that miR-495 inhibits cell migration in vitro and partly indicates the effect of miR-495 on the malignant phenotype of endometrial cancer.

### miR-495 downregulates FOXC1 expression by binding the 3′ UTR of FOXC1 in endometrial cancer

**Fig. 2:**
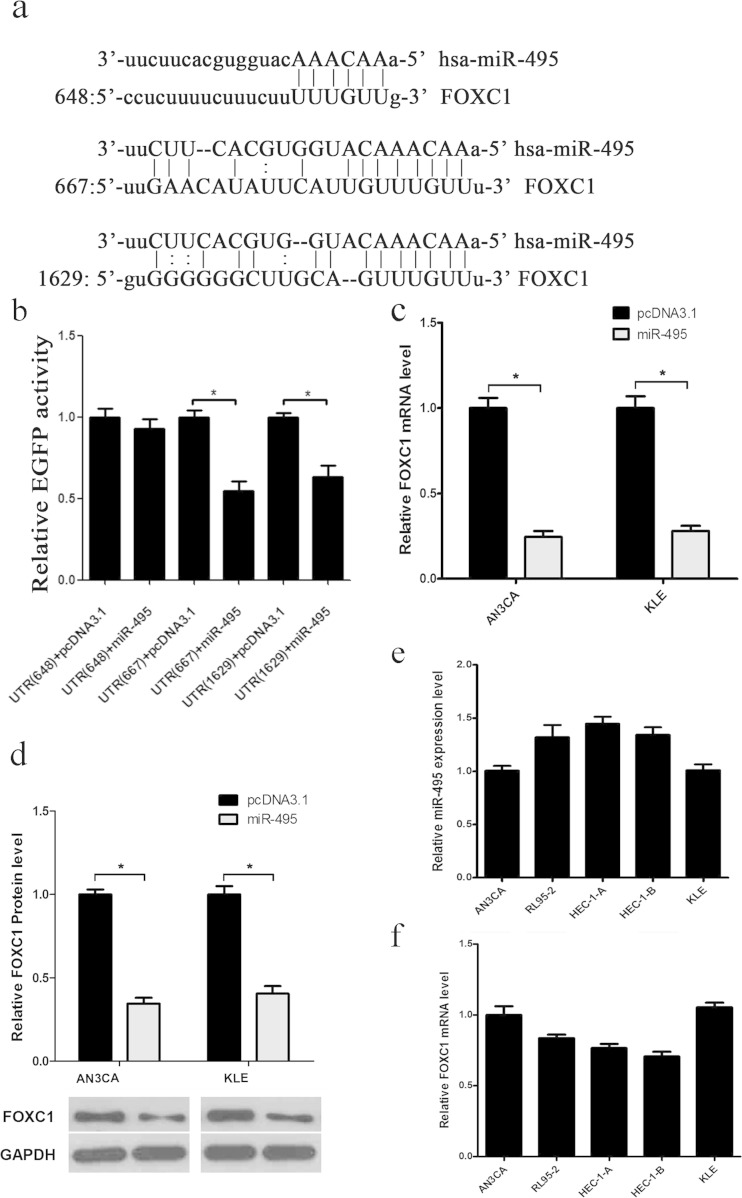
FOXC1 is directly repressed by miR-495. **a** The FOXC1 3′UTR has three putative miR-495 binding sites (648, 667 and 1269). **b** The intensity of EGFP fluorescence in reporter cells transfected with miR-495 was measured after 48 h of transfection. **c** The expression levels of FOXC1 in AN3CA and KLE cells were significantly altered following transfection with miR-495 by qRT-PCR using U6 snRNA for normalisation. **d** The protein levels of FOXC1 in AN3CA and KLE cells decreased when miR-495 was overexpressed. **e**, **f** The expression level of miR-495 and FOXC1 in five endometrial cancer cells (*p < 0.05)

The inhibition of cell growth and migration by miR-495 is derived from its target gene. To explore the mechanisms of miR-495 regulation in endometrial cancer, we searched for the potential targets of miR-495 using three bioinformatic algorithms: TargetScan, PicTar and miRanda. Using these programs, we selected FOXC1 as a miR-495 target gene for further study due to the malignancy phenotype in other types of cancer, which might be consistent with the phenotype of miR-495 in endometrial cancer. To elucidate whether miR-495 directly regulates FOXC1, an enhanced green fluorescent protein (EGFP) reporter assay was used to validate the target sites in the FOXC1 3′UTR. There are three predicted miR-495 binding sites (648, 667 and 1629) in the 3′UTR of FOXC1 mRNA (Fig. 2a). The binding sites for miR-495 on the FOXC1 3′UTR are conserved amongst species. First, we constructed an EGFP reporter plasmid by inserting the miR-495 binding sites (648, 667 and 1629) of FOXC1 3′UTR downstream of the EGFP stop codon (pcDNA3/EGFP- FOXC1-3′UTR 648, 667 and 1629). Next, AN3CA and KLE cells were cotransfected with the FOXC1-3′UTR reporter plasmid (UTR (648), UTR (667) and UTR (1629)) and pcDNA3.1/pri-miR-495 (miR-495) plasmids or a control vector. The fluorescent intensity was measured 48 h after transfection. As shown in Fig. 2b, the normalised intensity of fluorescence was significantly reduced in the pcDNA3.1/pri-miR-495 plasmid-transfected group in the sites of UTR (667) and UTR (1629) compared to the negative control. However, there was no significant difference in the normalised intensity of fluorescence cotransfected with FOXC1-3′UTR (648) and pcDNA3.1/pri-miR-495. These results suggested that miR-495 binds directly to sites 667 and 1629 of FOXC1-3′UTR.

In addition, to verify that miR-495 could downregulate the endogenous expression of FOXC1 at the post-transcriptional level, qRT-PCR and western blot analyses were performed to detect the effect of miR-495 on FOXC1 mRNA and protein expression. AN3CA and KLE cells were transfected with pcDNA3.1/pri-miR-495 (miR-495) and the negative control. As shown in Fig. 2c, the overexpression of miR-495 led to an approximate 75.5 % and 72 % reduction in FOXC1 mRNA expression in AN3CA and KLE cells, respectively. Additionally, the FOXC1 protein level was reduced by 65.5 % compared with the control group in AN3CA and KLE cells (Fig. 2d). These results suggested that miR-495 negatively regulates endogenous FOXC1 expression. Furthermore, real-time PCR was also performed in five endometrial cancer cell lines, and the opposite trend was observed in the miR-495 and FOXC1 mRNA expression, which is consistent with the regulation between them (Fig. 2e, f). In combination, miR-495 directly binds the 3′UTR of FOXC1 and suppresses its expression in vitro.

### FOXC1 promotes cell growth and migration and suppresses apoptosis in vitro

**Fig. 3:**
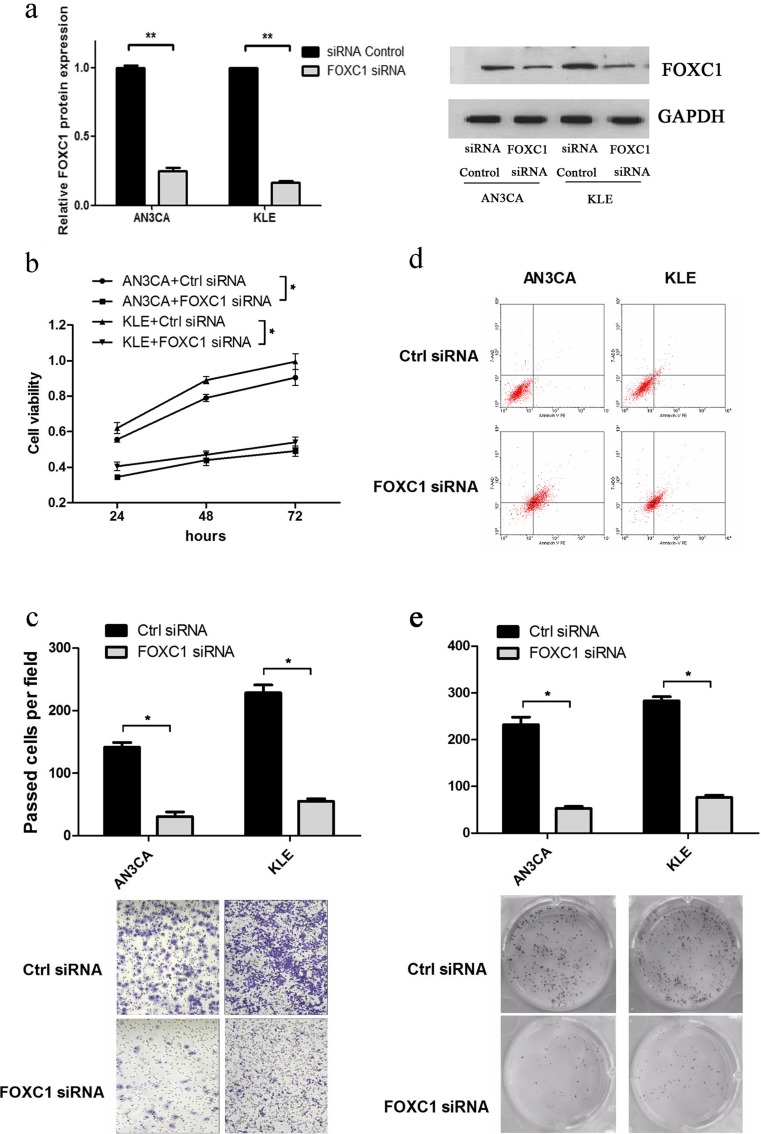
FOXC1 affects viability, colony formation apoptosis and migration in AN3CA and KLE cells. **a** Western blot was used the detect the expression of FOXC1 when FOXC1 was knocked down. **b** The cell viability of AN3CA and KLE cells was determined by MTT assay 24, 48 and 72 h after transfection (*n* = 3,*p < 0.05). **c** Transwell assay was employed to detect the effect of FOXC1 knockdown on the cell migration of AN3CA and KLE cells (*n* = 3,*p < 0.05). **d** Flow cytometry analysis was used to detect cell apoptosis in AN3CA and KLE cells. **e** Colony formation assay was employed to detect the effect of FOXC1 knockdown on the cell growth activity of AN3CA and KLE cells (*n* = 3,*p < 0.05)

Considering that miR-495 directly regulates the expression of FOXC1 and suppresses cell growth and migration in vitro, we next examined the function of FOXC1 in endometrial cancer. An siRNA expression vector (siFOXC1) was constructed successful (Fig. 3a) and transfected into AN3CA and KLE cells to test cell viability, colony formation, apoptosis and migration. As expected, siFOXC1 caused a significant reduction of cell viability in AN3CA cells using the MTT assay (Fig. 3b). Similarly, the inhibition of FOXC1 reduced cell viability in KLE cells (Fig. 3b). Meanwhile, a colony formation assay was performed to determine the long-term effect on cell growth. The formation of colonies in the group of FOXC1 siRNA was reduced approximately 77.16 % and 72.97 % in AN3CA and KLE cells (Fig. 3e). Next, we performed FCM to examine the properties of cell apoptosis. As shown in Fig. 3d, cells transfected with FOXC1 siRNA had a higher rate of cell apoptosis compared with the negative control both in AN3CA cells and KLE cells, which indicated that the contribution to cell growth by FOXC1 was due to the inhibition of apoptosis. Furthermore, we also performed a transwell migration assay to determine the effect of FOXC1 on cell migration in endometrial cancer. Knockdown of FOXC1 expression resulted in a significant decrease in the rate of cell migration in AN3CA (78.45 %) and KLE (75.93 %) cells compared with the negative control (Fig. 3c). These results demonstrated that the promotion effect on cell growth and migration by FOXC1 is consistent with the effect of miR-495 in endometrial cancer.

### Ectopic expression of FOXC1 counteracts the effects of miR-495 in endometrial cancer cells

**Fig. 4:**
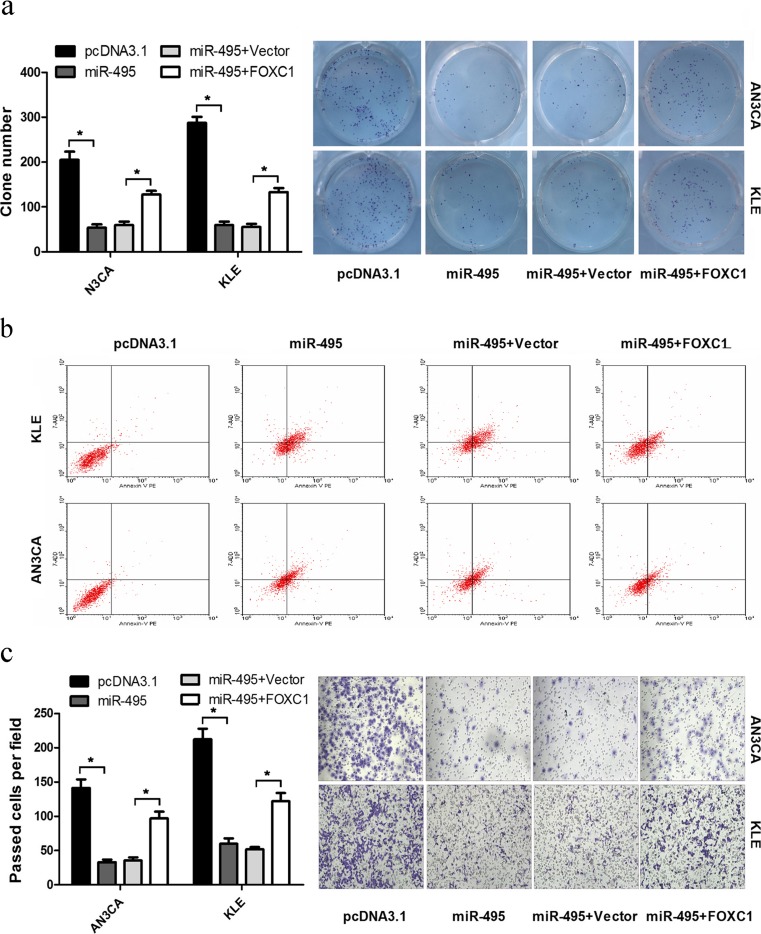
FOXC1 rescues miR-495-induced cellular phenotypes in endometrial cancer. **a** A colony formation assay was performed after transfection. **b** Flow cytometry analysis was used to detect cell apoptosis in AN3CA and KLE cells. **c** The ectopic expression of FOXC1 without a 3′UTR rescued AN3CA and KLE cell migration (*p < 0.05)

The previous results confirmed that the overexpression of miR-495 induced the inhibition of cell growth and migration required by downregulation of FOXC1. To further confirm that the effects of miR-495 on the growth and migration of AN3CA and KLE cells are mediated by FOXC1, a rescue experiment was performed. The overexpression vector of FOXC1, which contains the FOXC1 ORF without the 3′UTR, was constructed to avoid the influence of miRNAs. If the effect of miR-495 is specific, co-expression of FOXC1 should be able to reverse the phenotype of miR-495 overexpression. The AN3CA and KLE cells were cotransfected with pcDNA3.1/miR-495 plus pcDNA3.1/FOXC1 or pcDNA3.1/miR-495 plus empty pcDNA3.1. In the colony formation assay, ectopic FOXC1 expression counteracted the inhibition of cell growth and migration caused by miR-495 compared with the control vector (Fig. 4a). As shown in Fig. 4b, the FCM assay indicated that the overexpression of FOXC1 reversed the promotion of miR-495 on apoptosis on AN3CA and KLE cells. In addition, migration caused by miR-495 was abrogated in cells cotransfected with the FOXC1 ectopic vector (Fig. 4c). Therefore, these results provide further evidence that FOXC1 functions as a target of miR-495 and is involved in the miR-495-mediated malignancy phenotype of endometrial cancer.

### miR-495 suppressed tumour growth in vivo

**Fig. 5:**
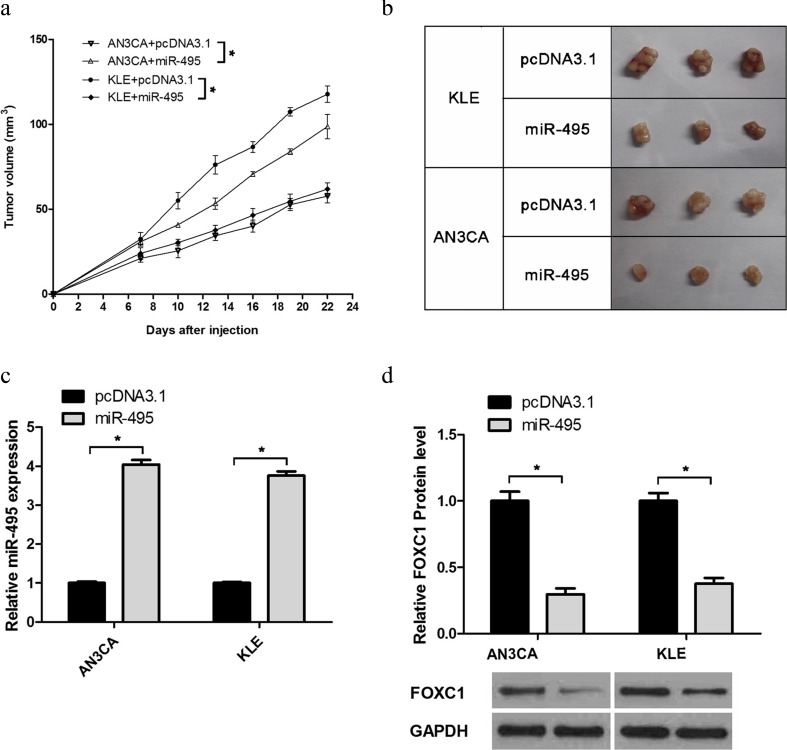
miR-495 suppresses the growth of endometrial cancer through the downregulation of FOXC1 in vivo. **a** The average volume of the tumour nodules derived from AN3CA and KLE cells that were stably expressed miR-495 in SCID mice. **b** The nodules obtained from the cancer cells injected subcutaneously into the flanks of nude mice. **c**, **d** qRT-PCR and western blot assay were performed to evaluate the expression levels of miR-495 (**c**) and FOXC1 (**d**) in the tumour nodules (*p < 0.05)

To examine the effect of miR-495 on the growth of tumours in vivo, we screened AN3CA-pooled clones and KLE-pooled clones with G418 to gain the stable expression of miR-495 (AN3CA/miR-495, KLE/miR-495) and the negative control (AN3CA/pcDNA3.1 and KLE/pcDNA3.1). AN3CA/miR-495- or AN3CA/pcDNA3-pooled clones were injected subcutaneously into the flanks of nude mice. The volume of the nodules was measured every three days starting from the seventh to the 22nd day to examine the effect of miR-495 on tumour growth. Compared with the control group, the volume of nodules was smaller in the AN3CA/miR-495 group than in the AN3CA/pcDNA3.1 group (Fig. 5a). The KLE-pooled clones obtained the same results (Fig. 5a). After 22 days, the nodules were harvested and divided for qRT-PCR and western blot analyses. The volume of nodules was smaller in the miR-495 both in AN3CA- and KLE-pooled clones (Fig. 5b). qRT-PCR was used to examine the expression of miR-495 in the pooled clones. As expected, the miR-495 expression was increased (75.25 % and 73.40 %) in the nodules of miR-495 overexpression derived from AN3CA- and KLE-pooled clones (Fig. 5c), respectively. To further determine the relationship between miR-495 and FOXC1 in vivo, we performed western blotting to examine FOXC1 expression. The results demonstrated that the expression levels of FOXC1 in the AN3CA/miR-495 and KLE/miR-495 groups were reduced by approximately 70.5 % and 62.50 %, respectively, compared with the negative control (Fig. 5c), which were consistent with the results from endometrial cancer cells in vitro. Together, these results indicated that miR-495 suppresses the endometrial cancer growth and downregulates the expression of FOXC1 in vivo.

### Expression of miR-495 and FOXC1 in endometrial cancer tissues and cells

**Fig. 6:**
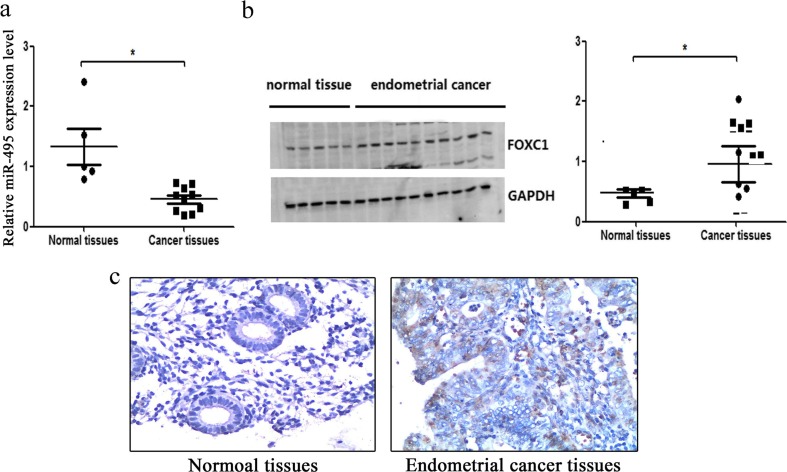
The expression of miR-495 and FOXC1 in endometrial cancer tissues. **a**, **b** qRT-PCR and western blot assay were used to determine the expression levels of miR-495 (**a**) and FOXC1 (**b**) in ten endometrial cancer tissues and five normal tissues. **c** The expression of FOXC1 in the endometrial cancer tissue and normal tissue was detected using immunohistochemistry (*s < 0.05)

To evaluate the potential role of miR-495 and FOXC1 in endometrial cancer, ten tissues from endometrial cancer patients and five endometrial tissues from healthy people were applied to detect the expression level of miR-495 and FOXC1 using qRT-PCR, western blot and immunohistochemical staining. We found that miR-495 expression decreased progressively both in endometrial cancer patients’ tissues and healthy people’s endometrial tissues compared with miR-495 expression from one normal tissue (Fig. 6a). However, miR-495 expression from endometrial cancer patients’ tissues decreased to a greater extent. Consistently, western blot assay indicated that the expression of FOXC1 in endometrial cancer patients’ tissues was much higher compared with the normal tissues (Fig. 6b). To further confirm the reduced levels of FOXC1 expression in endometrial cancer tissues, we utilized immunohistochemical staining to detect the FOXC1 protein levels. Compared with those of the normal tissues, the FOXC1 protein levels were markedly increased in the endometrial cancer patients’ tissues (Fig. 6c). Overall, these results confirmed the relationship between miR-495 and FOXC1 and their functions in endometrial cancer tissues.

## Discussion

Endometrial cancer is the most widespread gynaecologic cancer amongst women. According to the clinical, molecular and pathological data, endometrial cancer can be classified into two subgroups [12]. The endometrioid type (Type I tumours), which exhibits an altered PI3K/PTEN/AKT/mTOR signal pathway, is oestrogen-dependent and well-differentiated with a relatively favourable prognosis [26, 27]. The non-endometrioid type (Type II tumours) exhibits p53 mutations [28] and epidermal growth factor receptor 2 (HER-2) [29], is oestrogen-independent and has a serous prognosis [30]. In concert, aberrant molecular expression, including both oncogenes and tumour suppressors, has been linked to tumourigenesis and the progression of endometrial malignancies [31]. From a mechanistic perspective, several regulators of gene expression have been identified, in which miRNAs play a critical role. miRNAs have been described as oncogenes or tumour suppressors by regulating gene expression at the post-transcription level. Our study demonstrated the function of a tumour-related miRNA, miR-495, in regulation of endometrial cancer.

We provide several levels of evidence to identify miR-495 functions in regulation of endometrial cancer phenotypes. We used the MTT assay to determine the effect of miR-495 on cell viability in the AN3CA and KLE endometrial cancer cell lines. When miR-495 was overexpressed, the viability of endometrial cancer was reduced. In the colony formation assay, we observed that the colony formation activity of AN3CA and KLE cells transfected with pri-miR-495 was significantly inhibited. To further explore the mechanism of the viability and inhibition of colony formation by miR-495, we examined cell apoptosis and the corresponding molecular marker. The results showed that miR-495 could induce the apoptosis of AN3CA and KLE cells. Furthermore, the migration activity of AN3CA and KLE cells transfected with miR-495 was significantly decreased. In parallel, the animal study showed that miR-495 could repress the growth of nodules. These results highlight the significance that miR-495, acting as a tumour suppressor, alleviated the malignancy of endometrial cancer both in vivo and in vitro.

As is commonly known, miRNAs possess different roles by regulating target gene expression [32]. The identification of specific miRNA target genes is critical for understanding the mechanism involved in miRNA-related carcinogenesis. Increasing evidence has shown that miRNAs regulate their target genes by binding to the mRNA 3′UTR [33]. To identify the target gene responsible for the effects of miR-495 on endometrial cancer, we used bioinformatics and functional knowledge to predict the miR-495 target gene and chose FOXC1 as a candidate. The EGFP reporter system was used to confirm the miR-495 binding sites in FOXC1 3′UTR. Three sites exist in the FOXC1 3′UTR, and two of them are responsive to miR-495 overexpression. The EGFP fluorescence intensity of FOXC1 3′UTR was specifically reduced in the miR-495 overexpression group. These results suggested that miR-495 regulates FOXC1 expression by directly binding to the FOXC1 3′UTR. In parallel, endogenous FOXC1 mRNA and protein expression decreased both in AN3CA and KLE cells transfected with pri-miR-495. In addition, the inverse correlation between miR-495 and FOXC1 mRNA in endometrial cancer cells and tissues further supported this conclusion. Moreover, the expression of FOXC1 was reduced in the nodules, which had a high level of miR-495 in vivo. Taken together, these results confirm the direct regulation of miR-495 on its target FOXC1.

The FOX gene family belongs to transcription factors and is involved in many aspects of physiology and pathology [34]. FOXC1, which contains a characteristic DNA-binding forkhead domain (FHD), is a member of the FOX gene family [35]. Thus, FOXC1 also participated in the development of cancer by regulating the target genes. FOXC1 interacts with the Bmp-responsive enhancer to reduce Msx2 expression and contribute to skull vault growth in breast cancer [36]. MMP7 has a downstream effect on FOXC1-mediated invasiveness [37]. Meanwhile, increasing numbers of studies have shown that some genes could directly regulate FOXC1 expression. In breast cancer, EGFR activation induced FOXC1 transcription and FOXC1 knockdown impaired cell proliferation and migration [38]. FOXC1 is downregulated by miR-204 and reduces cell migration in endometrial cancer [17]. Our results reveal the link between miR-495 and the oncogenic factor FOXC1, because FOXC1 was confirmed to be a direct and functional target gene of miR-495 in endometrial cancer. Thereafter, we aimed to study whether the function of FOXC1 was consistent with this regulation. The effect of FOXC1 on the processes of endometrial cancer was validated by FOXC1 gain and loss of function followed by cell viability, colony formation, apoptosis and migration assays. In AN3CA and KLE cells, FOXC1 promoted cell viability and colony formation by reducing cell apoptosis. Previous studies showed that FOXC1 regulates the epithelial-mesenchymal transition (EMT) and contributes to cell migration [39, 40]. In this study, we also obtained the promotion of migration by FOXC1, which is consistent with this study. Importantly, the overexpression of FOXC1 can reverse the cell functions induced by miR-495 overexpression, indicating that miR-495 reduced the malignancy of endometrial cancer, at least partially, by regulating FOXC1.

In summary, miR-495 functions as a tumour suppressor gene and exhibits its biological role by regulating the expression of FOXC1 in endometrial cancer. Our study demonstrated a novel regulatory mechanism by which miR-495 inhibits endometrial cancer cell growth by inducing apoptosis and migration in vitro. Additionally, FOXC1 acts as an oncogene in endometrial cancer. Animal studies also confirmed the negative regulation of FOXC1 by miR-495 in vivo. This study helps us to further understand the miRNA-mediated regulation mechanism in endometrial cancer and may provide an important molecular diagnosis and treatment strategy for patients with endometrial cancer.
